# A Novel Approach of Bioesters Synthesis through Different Technologies by Highlighting the Lowest Energetic Consumption One

**DOI:** 10.3390/polym13234190

**Published:** 2021-11-30

**Authors:** Simona Popa, Andra Tamas, Vasile Simulescu, Dorin Jurcau, Sorina Boran, Giannin Mosoarca

**Affiliations:** 1 Faculty of Industrial Chemistry and Environmental Engineering, Politehnica University Timisoara, Bd. V. Parvan, No. 6, 300223 Timisoara, Romania; simona.popa@upt.ro (S.P.); andra.tamas@upt.ro (A.T.); 2 Faculty of Chemistry-Biology-Geography, West University of Timisoara, Bv. Vasile Parvan, No. 4, 300223 Timisoara, Romania; vasile.simulescu@e-uvt.ro; 3 Elkim Special SRL, Str. Constantin cel Mare, No. 21, 300265 Timisoara, Romania; lk-eldus@rdstm.ro

**Keywords:** soybean oil fatty acids, bubble column reactor, microwave reactor, lowest energetic consumption, color, rheology

## Abstract

Fatty acids esters have a wide application as bioplasticizers and biolubricants in different industries, obtained mainly in classic batch reactors, through an equilibrium complex reaction, that involves high temperatures, long reaction times, vigorously stirring, and much energy consumption. To overcome these shortcomings, we synthesized a series of fatty acid esters (soybean oil fatty acids being the acid components with various hydroxyl compounds) through novel low energy consumption technologies using a bubble column reactor, a microwave field reactor and for comparison meaning, a classic batch reactor. The obtained bioesters physicochemical properties were similar to one another, a good concordance among their rheological properties was obtained, but the energetic consumption is lower when using the bubble column or the microwave reactors instead of the classical batch reactor.

## 1. Introduction

Environmental protection efforts in the industry is focused on reducing wastes by recycling some of these materials [1] and replacing the raw materials of petroleum-based products used in different industries with organic fluids [2,3]. At the same time, modern technologies should be friendly with the environment [4,5,6], obviously in terms of energy and economic efficiency. Industrial soybean oil is often used, by further processing, as an ingredient for paints, plastics, fibers, detergents, cosmetics and lubricants that show similar viscosity to commercial lubricants [7]. Natural solvent and for example, clear liquid soy derived from soybean oil as methyl esters could serve as a green alternative to synthetic solvents. In addition, due to their low evaporation rate and longer contact time, maintained with a target material, they are recommended as natural adjuvant and surfactant in order to increase crop yields while lowering input costs by improving contact of spray droplets on plant surfaces and more effectively penetrating waxy surfaces. Soy esters present some benefits as safe to handle and store, low toxicity compared to other common substances. Vegetable oils, containing non-toxic and ecofriendly fatty acids, are successfully used by esterification and transesterification syntheses in biodiesel production [8,9,10], which is one of the main important biodegradable products [2,11,12,13,14,15]. Color properties of certain substances is often used in describing their properties, either organic or inorganic ones, as well as in food field [16,17,18]. As known, rheological behavior is important because it provides information regarding flow and storage of relevant materials under operation conditions [19] especially when the products are used in paint industry. Rheology is used in many researches, as for the analysis of engine oil lubricants [16,17,18,19,20,21,22,23,24], hydrogels [25,26], different polymer—plasticizer systems [27,28], heterocycles [29,30,31], nanofluids [32,33] collagen solutions [34], cyclodextrin nanospongies [35] and other. Energy efficiency represents another important issue in today’s technology, in order to find and implement low cost processes [36,37]. At present, the esterification processes in classical batch technology require much energy consumption, because of the necessity of high temperatures (up to 220–250 °C), long reaction time, and vigorously stirring. That is why researches are made to find new energy-saving technological methods [12,13,37].

At present, the microwave heating is gaining more and more influence in technological processes due to its energy economy and environmental advantages, being used in different fields [38,39,40,41,42,43,44].

To overcome these shortcomings, the present paper refers to the synthesis of a series of fatty acid esters via two modern technologies that do not deal with solvent extraction of the azeotrope namely a bubble column reactor [11,45] and a microwave field reactor. In this reactor the process time is short, microwave heating being a widely accepted tool for synthetic chemists [26,46]. For the sake of comparison, the synthesis was performed in a classic batch reactor as well. To the best of our knowledge, there is no reported work on a comparison between energy consumption in the various esterification reactors, or a comparative rheological study of the obtained products. Therefore, this paper investigated these aspects, revealing that the bubble column reactor and the microwave field reactor are energy-saving technological methods for the bioester synthesis. All products properties are similar, regardless the synthesis method.

## 2. Materials and Methods

The soybean oil fatty acids were received from Baichim SRL Bucharest. The organic alcohols n-propanol, n-butanol and n-pentanol, and the catalyst p-toluene-sulfonic acid were purchased from Fluka Honeywell (Charlotte, NC, USA). The fatty acids (R-COOH) from hydrolyzed soybean oil have a typical composition containing 11% palmitic acid, 4% stearic acid, 25% oleic acid, 50% linoleic acid and 9% linolenic acid. The physicochemical properties of fatty acids from used soybean oil are: viscous liquid without mechanical impurities; yellow color; 0.89 g/cm^3^ density at 20 °C; 14–16 °C melting point; 193.4 mg KOH/g acid number; −1.458 refraction index at 20 °C.

The main esterification reactions were performed in a bubble column reactor (B), in a microwave reactor (M) and in a classic batch reactor (C), using soybean oil fatty acids as the acid component and three hydroxyl-compounds: n-propanol (1), n-butanol (2), and n-pentanol (3), with the 1:2 mole fraction between the fatty acids from soybean oil and the organic alcohols respectively. The catalyst p-toluene-sulfonic acid was used in proportion of 0.4%. The obtained bioesters with n-propanol are B1, M1 and C1, with n-butanol are B2, M2, C2, and n-pentanol are B3, M3, C3 respectively.

A cylinder glass column with an internal diameter of 0.03 m and a height of 0.3 m provided with a heating mantle was used as bubble column reactor. Agitation was achieved by bubbling nitrogen through a nozzle at the base of the column. The energetic efficiency calculation of the bubble column reactor was developed upon esterification reaction among benzoic acid and propylene glycol, using different reaction conditions [47]. Considering the best conditions achieved in terms of energy consumption (the nozzle of 0.6 mm, argon pressure of 123.6 Pa and with no filling material) esterifications in the bubble column reactor were carried out. Esters of soybean oil fatty acids with the organic alcohols were synthesized in two steps. The first step took place in a flask fitted with a thermometer and a water reflux cooler, where the preheating of the reaction mixture was carried out for one hour under continuous stirring, at 60 °C on an electric stove of 5 kW. Then the product was transferred directly into the column reactor, where the synthesis was carried out at the reflux temperature, to give the products B1, B2 and B3. The reaction conditions and times are presented in Table 1.

Fatty acid esters, namely M1, M2 and M3 were obtained in a chemical reactor with a microwave heating oven (model DB-001, China Doble Best, China) provided with a reflux cooler, in a single step in the presence of the catalyst—p-toluene sulfonic acid at the reflux temperature (Table 1). The characteristics of the chemical reactor with microwave (M) heating are: microwave power 0~800 W; microwave frequency 50 MHz 2450+; Shaking magnetic stirrer.

In the classic technology, the reactor (Model ELN9.1, Carl Roth GmbH + Co. KG, Karlsruhe, Germany) was heated with an electric stove of 5 kW. The synthesis was performed in a solution esterification process, using p-toluen sulfonic acid as catalyst. The water azeotrope was extracted with toluene. The esterification was carried out in a single step according to the reaction conditions presented in Table 1, with the formation of products C1, C2 and C3.

In all cases, the esterification was monitored by periodic determination of the acid number over the whole synthesis, and the process was considered to be completed when acid index (IA) was below 1 mg KOH/g. The synthesized esters were purified by neutralization with 10% aqueous sodium carbonate solutions, washed with demineralized water to neutral pH, then vacuum distillation and decolourisation with activated charcoal and filtration. The purified compounds were then subjected to specific analysis.

The physicochemical properties of the bioesters were determined by using standardized techniques: density—SR EN ISO 12185-03, refractive index—SR 7573-95, acidity index—SR EN ISO 660:2009, iodine value—SR EN ISO 3961:2013, color—visual, rheological study was performed using a Brookfield CAP2000+L viscometer (AMETEK GmbH/B.U. Brookfield, Dresden, Germany), according to ASTM D445, temperature range 5–70 °C.

The calculated data are the mean of three independent replicates. Before running the one-way ANOVA analysis, Equal Variances tests (Multiple comparisons and Levene’s methods) were performed to verify that the samples have equal variances. The 95% confidence level was adopted and the Tukey pairwise comparisons was applied to establish the significant differences between samples. Minitab 19 software (Minitab, LLC, USA) was utilized to perform the required calculations.

Thermo-gravimetric (TG/DTG) analyses were performed with NETZSCH STA 449F1 STA449F1A-0220-M (NETZSCH-Gerätebau GmbH, Germany)—approximately 3–7 mg of sample was heated in an Al_2_O_3_ crucible, with 5 °C/min, in nitrogen atmosphere, within the range 20–600 °C. An ion trap mass spectrometer ITQ 1100 coupled with Gas Chromatograph Trace 1310 (Thermo Fisher Scientific, Waltham, MA, USA) was used for qualitative analysis of soybean fatty acids bioesters. MS parameters were set as following: transfer line temperature at 310 °C, source temperature at 170 °C and scan range between 30 and 350 amu. The reaction product structure was established based on the *m*/*z* ratio. Fourier Transform Infrared (FT-IR) spectra of the samples were obtained in attenuated total reflectance (ATR) mode on a Bruker Vertex 70 (Bruker Daltonik GmbH, Bremen, Germany) spectrometer equipped with a Platinum ATR, Bruker Diamond Type A225/Q. Spectra were collected in the range 4000–400 cm^−1^ with a resolution of 4 cm^−1^ and with 40 scans/min. A MINOLTA CM 3220d spectrophotometer (Konica Minolta Sensing Europe B.V., Nieuwegein, The Netherlands) was used for the colorimetric analysis of the applied pigment in films in the following conditions: the CIE D65 illuminant (natural day light) and the standard 10° observer function.

## 3. Results and Discussion

In the present study, three esterification methods were used for obtaining of bioesters from soybean fatty acids with different alcohols. Raw materials are well mixed and esterification reactions can be completed within 3 to 10 h depending on esterification method. The aim was to determine which synthesis method is more energy efficient. As it was expected, microwave and bubble column methods are promising routes with lower energetic consumption.

### 3.1. Energetic Efficiency Comparison

Taking into account the properties of the heating devices used in the three technologies of this research, the calculated energy consumption of the process developed in the bubble column reactor was approximately 18,000 kJ for one synthesis of 300 g bioester. In the reactor with a microwave field the energy consumption for the same esterification process was approximately 8700 kJ, and in the classical reactor 144,000 kJ. As can be seen, for the same bioester production, the use of the classical reactor requires a much more energy consumption than in the case of the other two technologies, which are also environmental protective ones.

### 3.2. Bioesters Analysis

**Figure 1:**
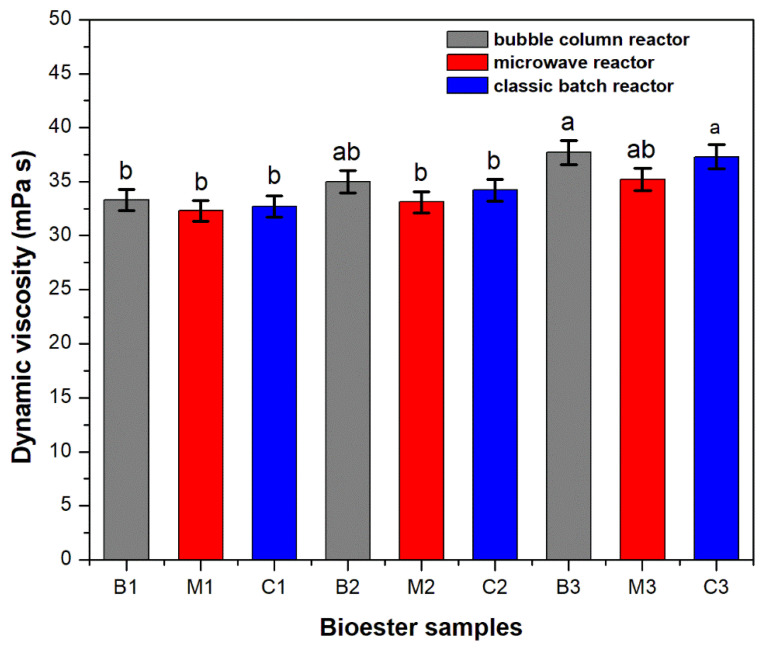
Dynamic viscosity at 25 °C, for the synthetized bioesters. Values are expressed as means of three independent replicates and error bars represent the standard deviation. Columns denoted by different letters indicated significant (*p* < 0.05) differences among different synthesis conditions.

In order to verify the properties of the esters of soybean oil fatty acids with various alcohols synthesized via the technologies mentioned above, the purified products were characterized by physicochemical analyzes specific to this class of substances (Table 2 and Figure 1).

All products showed viscous, opalescent aspect, with a light brown-orange color. The unsaturated degree of oils being appreciated by the iodine index, the iodine values of the synthesized bioesters are in the range of 120–140 g I_2_/100 g, according to the one of the soybean oil. As known, acidity is an indication of the presence of free fatty acids that are to be limited due to the formation of soaps, which may lead to the emulsions formation. The values of the acid index indicate a low content of free fatty acids. The values of the refractive index and density at 20 °C, presents a slightly variation. The dynamic viscosity increases with the number of carbon atoms brought by the alcoholic rest. Similar results were reported in the literature [8].

The results from Figure 1 show that there was no statistically significant difference of this parameter between esters, obtained with n-propanol (B1, M1, C1), synthetized in bubble column reactor, microwave reactor and classic batch reactor. The same conclusion can be drawn by observing the results concerning the esters obtained with n-butanol (B2, M2, C2) and n-pentanol (B3, M3, C3) respectively.

All that suggested the formation of similar esters despite the different reactors used for their synthesis.

### 3.3. TG/DTG Analysis

Thermo-gravimetric analysis provides essential information on the relative thermal stability of the analyzed compounds, the content of water or other volatile ingredients of synthetic or natural materials [48]. From thermo-analytical curves, recorded for the synthesized esters in the range of 25 and 600 °C in nitrogen atmosphere, the inflection points and the total weight loss at 600 °C (Table 3) indicate that the method used does not affect the physicochemical properties of the obtained esters.

All of the obtained bioesters present low weight loss until 200 °C (lower than 3.5%). Therefore, the products exhibited good thermal stability below 200 °C so they may be used in technologies where such property is required, for example as natural adjuvant and surfactant in increasing crop yields with lowering costs. Above 300 °C, the weight loss is more important as the temperature increases.

For all studied bioesters the total weight loss appears around 600 °C, and the percentage of the residual mass remaining is insignificant. Three decomposition steps were observed. The first step corresponds to the water loss, are max. 3.5% (Table 3).

The observed mass loss values are similar and are not affected by the nature of the synthesis method. The second decomposition step (between 210 °C and 350 °C) and the third step (between 350 °C and 550 °C) were associated with the destruction of esteric group, the C-C and the C-H bonds. The decomposition process presents two inflection points at T_1_ and T_2_, associated with the highest decomposition rates.

Nitrogen atmosphere thermal stability analysis of methyl and ethyl esters of soybean oil [49] showed that they have a lower thermal stability compared to the samples synthesized by us by the three obtaining methods.

Comparable results were also obtained for erythritol tetra myristate and erythritol tetra laurate esters [50] or lubricants of the type gallate ester oils, respectively [51].

In contrast the polyester amides series [52] and the esters obtained by transesterification of palm oil-based methyl ester to trimethylolpropane esters [53] show better thermal stability compared to our samples.

**Figure 2:**
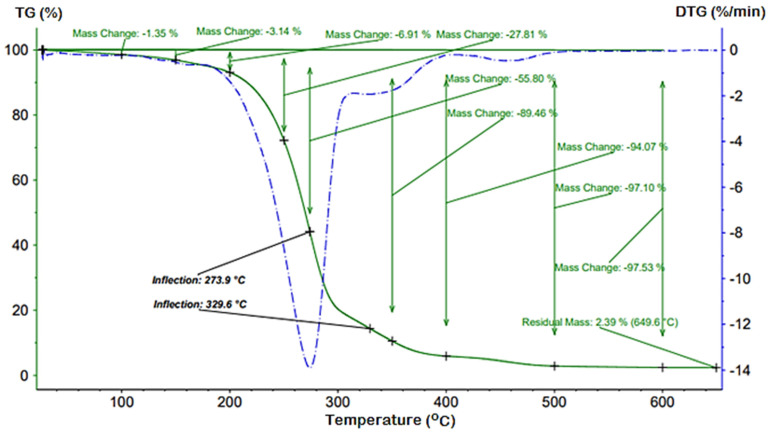
TG/DTG curves of bioester B3, in nitrogen atmosphere.

Figure 2 presents an example of the degradation process of the B3 product, in nitrogen atmosphere.

### 3.4. GS-MS Analysis

**Figure 3:**
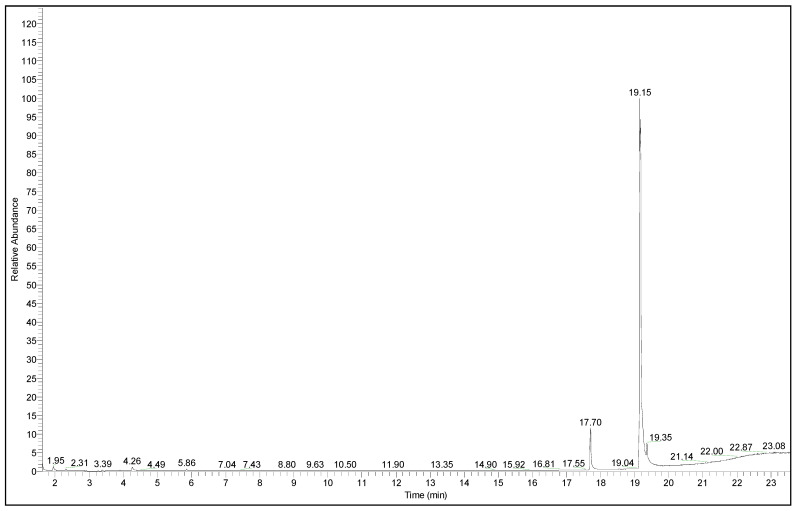
The chromatogram of the reaction mixture resulted from the esterification reaction in the case of B3 bioester.

**Figure 4:**
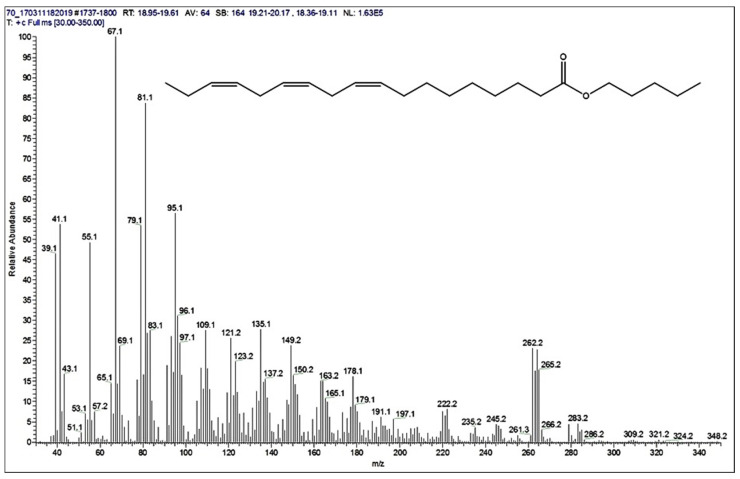
The mass spectrum of the ester, corresponding to the peak separated on the GC column at 19.15 min retention time (M = 348 g/mol), in the case of B3 bioester.

**Figure 5:**
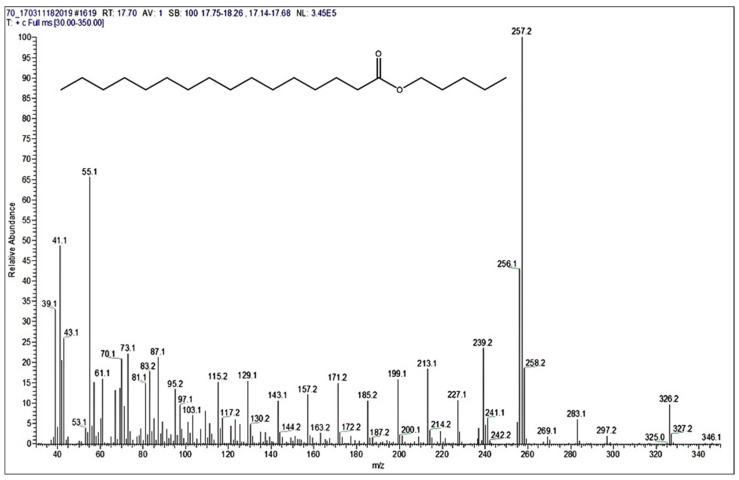
The mass spectrum of the ester, corresponding to the peak separated on the GC column at 17.70 min retention time (M = 326 g/mol), in the case of B3 bioester.

GS-MS spectra (Figure 3, Figure 4 and Figure 5) are also presented for the B3 bioester. The chromatogram indicates the presence of two main reaction products. The major compound was separated in the analysis conditions at the retention time of 19.15 min. Its peak had the highest intensity from the whole GC chromatogram. The second reaction product, pentyl palmitate, was eluted in the GC chromatogram at the retention time of 17.70 min (Figure 3).

In the Figure 4, the molecular peak at *m*/*z* 348, as expected for the main product, was observed with very low intensity, mainly due to its fragmentation. From the MS spectrum it is obvious that the structure of the analyzed compound that the structure is most probably (9Z,12Z,15Z)-octadeca-9,12,15-trienoate. This is proved because the first fragmentation of this compound is expected to occur at carboxyl group, resulting in the signal observed at 262.2 *m*/*z*. Therefore, this signal belongs to the fragment remained after losing thepenthoxy group.

Furthermore, on the region of lower mass values, several differences of around 14–15 *m*/*z* units were observed. This proved that CH_3_ and/or CH_2_ fragments are present, either from penthyl or from octadeca-9,12,15-trienoate or even from both.

The MS spectrum of the compound which eluted first (see Figure 3) at 17.7 min, but with a lower intensity, is presented in Figure 5. From this MS spectrum (Figure 5) the molecular peak at 326 *m*/*z* can be easily observed, with a better intensity. Moreover, in the same mass spectrum presented in Figure 5, the signals observed at 257.2 and 239.2 *m*/*z* indicated the presence of palmitate fragments. Nevertheless, the peak observed at 257.2 *m*/*z* showed also the highest intensity from the whole MS spectrum of pentyl-palmitate.

This proved that palmitate fragment had a higher ionization in the used ion source, in comparison with the other fragments formed during MS analysis. The signal obtained at 87.1 *m*/*z* proved the presence of penthoxy fragment, as for the previous analyzed ester. Moreover, in this region of the mass spectrum, were also observed more signals, at 71 *m*/*z*, 70 *m*/*z* and 69 *m*/*z* respectively. All of those peaks belong to pentyl fragments.

### 3.5. FTIR Analysis

**Figure 6:**
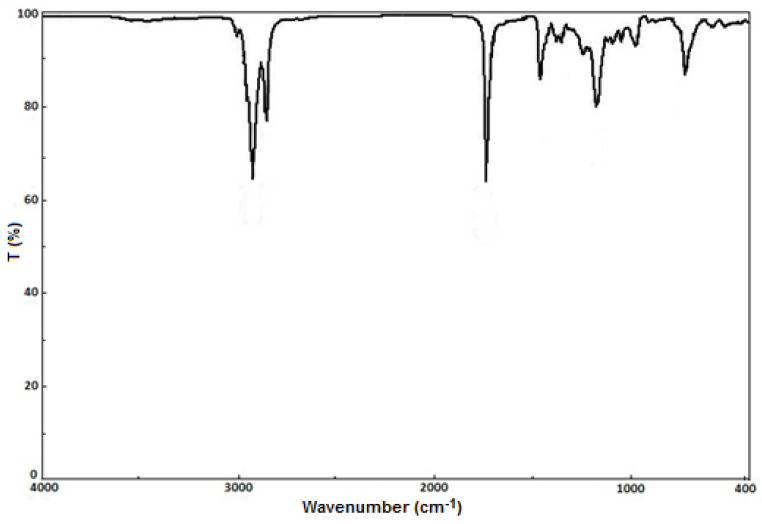
FT-IR analysis of the bioester B3.

The FTIR is often used in polymer analysis [54,55]. The FTIR spectrum of the bioester B2 (Figure 6) reveal peaks at 2926 cm^−1^ and 2855 cm^−1^. Those peaks can be attributed to the stretching of C-H bonds. At 1736 cm^−1^ the peaks are attributed to the stretching of C=O, typical of esters spectra [56]. The =C–H and C=C bands appear at 3012 cm^−1^ and 1657 cm^−1^. The peak at 1465 cm^−1^ correspond to the asymmetric stretching of -CH_3_ present in the biodiester. This peak is absent in soy oil spectrum. The peak at 1358 cm^−1^ was attributed to the O-CH_2_ group and the peak at 1173 cm^−1^ is corresponding to the stretching of O-CH_3_. Nevertheless, the peak at 1049 cm^−1^ was to attributed in plane deformation vibration of =C-H bond and the peaks at 926 cm^−1^ and at 725 cm^−1^ are attributed to the C–H wagging bond vibration. All of these proved also the absence of alcohol impurities.

### 3.6. Color Study

**Figure 7:**
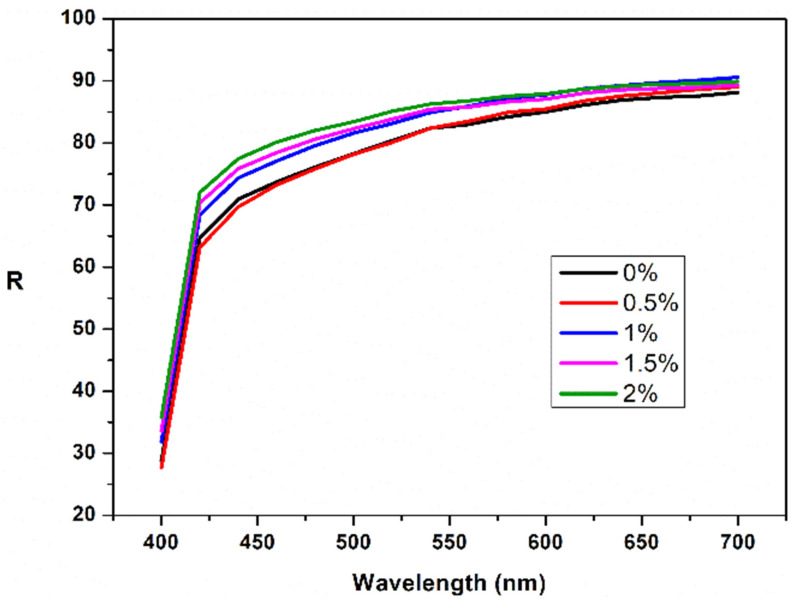
Reflectance spectra of the bioester B3 of the ester acrylic film at different ester concentration.

For the bioester B3 color study was also performed. The ester was introduced in different concentrations (0.1–2%) in an acrylic resin—that is often used in film industry [57,58]. The film was then deposited on a cellulosic support (wood). Color properties reveal that the reflectance increased with the ester concentration (Figure 7) according to other studies [59,60].

For all dried films, color CIEL*a*b* parameters were determined: lightness (L*), redness (a*), yellowness (b*), chroma or saturation (C*) and hue angle (h*) respectively.

**Figure 8:**
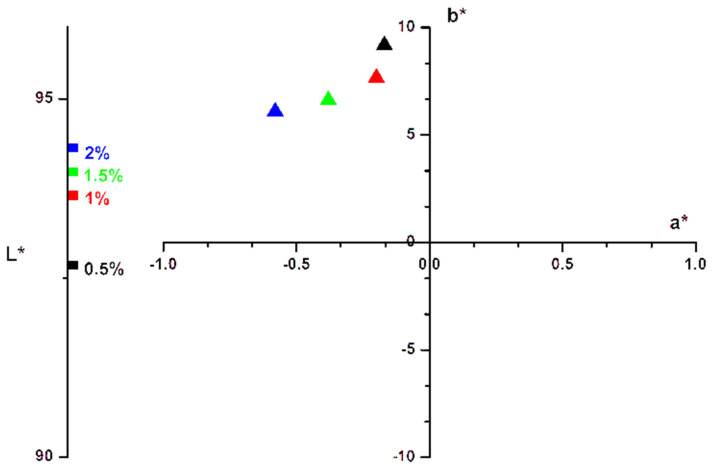
CIEL*a*b* properties of the bioester B3 of the ester acrylic film at different ester concentration.

The CIEL*a*b* color properties (Figure 8) reveal that the ester acrylic composition is in the light yellow-olive domain, i.e., the a* parameter is in the light green domain and the b* parameter is in the yellow domain. As expected, the film darkness increasing with the ester concentration. The same result were presented in scientific literature [18,60,61].

The total color difference ΔE*_ab_ may be calculated with the Equation (1) [17,62,63], results being presented in Table 4.

(1) $$\Delta E_{ab}^{*}=\sqrt{{\left(\Delta L^{*}\right)}^{2}+{\left(\Delta a^{*}\right)}^{2}+{\left(\Delta b^{*}\right)}^{2}},$$

### 3.7. Rheology Comparative Study of the Bioesters

**Figure 9:**
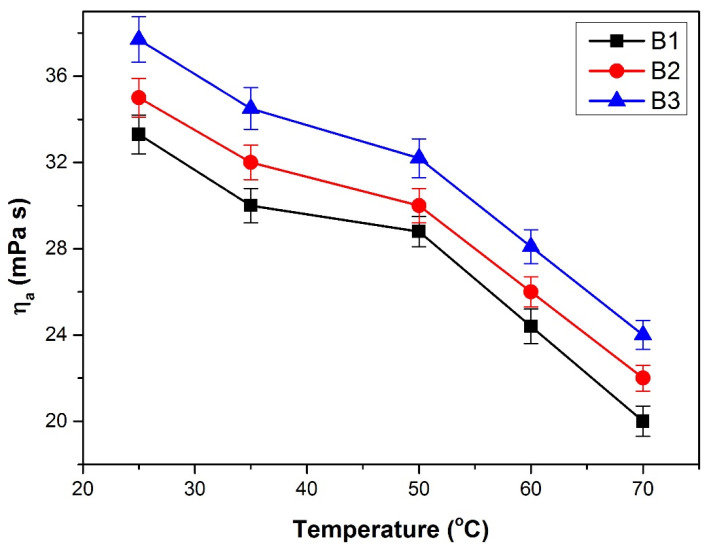
The dependence of the apparent viscosity on temperature for bioesters (B1–B3).

An important parameter that can remarkably influence the rheological properties of all fluids is temperature. Rheology measurements for all esters obtained in this research reveal a non-Newtonian behavior at the temperatures where their viscosity was measured (25–70 °C) and at different share rates (1333–13,333 s^−1^) [64]. The characteristic equation of Ostwald de Waele model (Equation (2) may be used for interpretation. All bioesters submitted to the rheology tests present a decrease of viscosity when increasing temperature. The same behavior was reported in other papers [18,65,66]. Although the allure of the curves is similar, the viscosity is higher as the number of the carbon atoms increases, exemplified for esters B1–B3 in Figure 9.

(2) $$\eta_{a}=K\gamma^{n-1},$$

where: *K*—the index of consistency, Pa·sn; *n*—the flow behavior index.

The pseudoplastic flow behavior is proved both by the decrease of the apparent viscosity with the increase of shear rate (shear-thinning behavior) [67], as well as by the sub-unit values of the flow index *n* (Table 5). The decreasing of the apparent viscosity with increasing temperature is also evidenced by the variation of the consistency index *k* correlated with the value of the flow index *n*.

**Figure 10:**
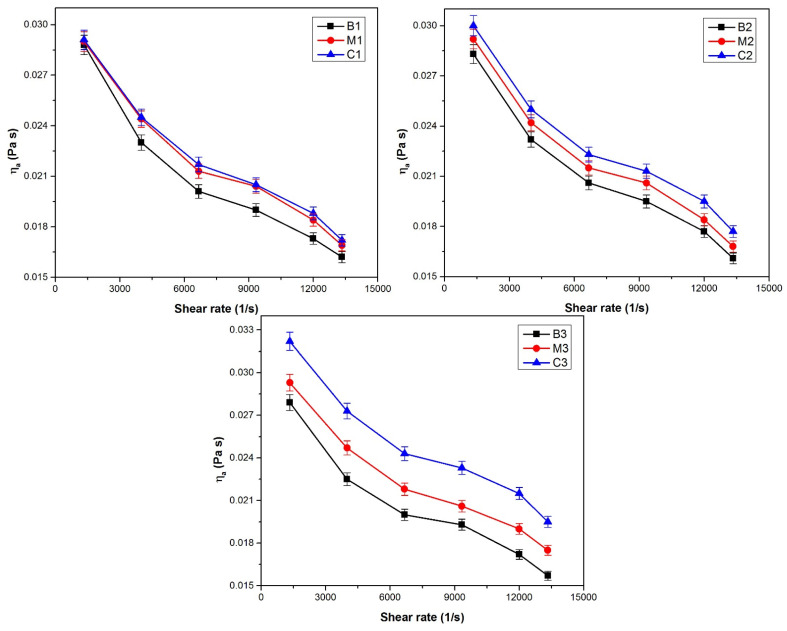
The dependence of the esters apparent viscosity on the share rate.

Power law model R^2^ values indicate that *k* and *n* values are a good fit. It was observed an increase of consistency coefficient (*k*) with the increase of carbon atoms number in alkyl group from alcohol. As expected, the increase in temperature reduces the consistency coefficient (*k*) and increase the flow behavior index (*n*) and at the same temperature, the decrease of consistency coefficient (*k*) is more evident for ester with long alkyl chain in alchool (B3). The modification of k with temperature revel some influence of Brownian movement. Regardless of the temperature and of the obtaining method, the apparent viscosities of the prepared with n-propyl alcohol (B1, M1, C1), at the same shear rate are very close to one another. For the esters prepared with *n*-butyl alcohol (B2, M2, C2) or *n*-pentanol (B3, M3, C3) the viscosities became more different, their values depending on the technology used (Figure 10).

They can act as thickening agents. According to rheological results, addition of bioester B2 as ingredient for paints, plastics, fibers, detergents, cosmetics and lubricants, is able to modify easily the rheological data and thicken the formulations. The temperature increase leads to micro drops mobility intensification, which influences the activation energy of the system. The phenomenon may be explained by an Arrhenius type equation (Equation (3)):

*η_a_* = *Aʹ* ∙ exp*^Ea/RT^*, (3)

where: *Ea* is the activation energy of viscous flow, (J/mol); *R* is the gas general constant, (J/mol∙K); *T* is absolute temperature and *Aʹ* represents the material constant, (Pa·s).

The dependence ln *η_a_* = f(*1/T*) was graphically represented, as obtained from the logarithmic form of Equation (3), for apparent viscosity values corresponding to the three chosen values of the shear rate. Particular expressions of Equation (3) as well as the values of the activation energy are presented in Table 6. The activation energy decreases with the increasing of the shear rate, due to the increase of the turbulence and its effect on the linearization tendency of the molecules, considering the reduction of the degree of association of the molecules as well. In both cases, the effort to move molecules is diminished. The activation energy of the bioesters varies in opposite direction with their viscosity when increasing the number of the carbon atoms in alcohols (Figure 10 and Table 6).

## 4. Conclusions

The synthesis of a series bioesters, using soybean oil fatty acids as the acid component, through three different technologies (in a bubble column reactor, in a reactor heated in a microwave field, and in a classic batch reactor) was performed. Energetic evaluation of the processes pointed out that the processes in the microwave field and in the bubble column reactor are more energetic efficient then in the classic batch reactor. The physicochemical and thermal properties of all esters were determined, and they present similar properties, regardless of the used synthesis routes. Rheological comparative study shows a pseudoplastic behavior for all esters. Equations of dependence of share stress on share rate, and of apparent viscosity on 1/T are proposed. Activation energy was determined for all samples and revealed an opposite variation with the bioesters viscosity when increasing the number of the carbon atoms in the alcohols from the constitution of esters. It should be noted that the results obtained herein can contribute to the development of new applications containing esters or to the synthesis of biopolymers using the low energy consumption and environmental friendly technologies.

## Figures and Tables

**Table 1:** The reaction conditions for soybean esters production in different technologies.

	Bubble Column			Microwave Field			Classic Reactor		
Bioester	B1	B2	B3	M1	M2	M3	C1	C2	C3
Reaction time, h	3	4	4	2	3	3	9	9	10
Temperature, °C	100	105	110	110	115	120	120	125	130

**Table 2:** The properties of esters via the technologies: bubble-column reactor (B1–B3), microwave heated reactor (M1–M3) and classical reactor (C1–C3).

Sample	Aspect	Color	Acid Index (mg KOH/g)	Refractive Index at 20 °C	Density at 25 °C (g/cm^3^)	Iodine Value (g I_2_/100 g)
B1	viscous, opalescent	orange	<1	1.4562 ± 0.0578	0.8981 ± 0.0271	120 ± 3
B2	gelatinous, opalescent	orange	<1	1.4577 ± 0.0581	0.8926 ± 0.0268	121 ± 4
B3	gelatinous	red-brown	<1	1.4591 ± 0.0584	0.9122 ± 0.0272	121 ± 4
M1	viscous	orange	<1	1.4499 ± 0.0575	0.8673 ± 0.0261	121 ± 3
M2	viscous	orange	<1	1.4598 ± 0.0587	0.8835 ± 0.0264	121 ± 5
M3	viscous	orange	<1	1.4611 ± 0.0584	0.9254 ± 0.0278	121 ± 4
C1	viscous, opalescent	orange	<1	1.4590 ± 0.0581	0.8810 ± 0.0263	120 ± 3
C2	viscous, opalescent	orange	<1	1.4593 ± 0.0579	0.8820 ± 0.0265	121 ± 5
C3	viscous, opalescent	orange	<1	1.4597 ± 0.0584	0.8815 ± 0.0264	121 ± 3

**Table 3:** The inflection points in the TG analysis.

Sample	T_1_ the Inflection Points (°C)	T_2_ the Inflection Points (°C)	Total Weight Loss at 200 °C (%)	Total Weight Loss at 600 °C (%)
B1	267	319	3.38	97.55
B2	274	330	3.21	97.61
B3	285	335	3.26	98.66
M1	263	315	2.96	98.77
M2	275	329	3.43	98.91
M3	282	333	3.13	97.64
C1	269	321	3.36	99.11
C2	277	330	2.98	98.98
C3	286	342	3.02	97.93

**Table 4:** Color differences of the ester acrylic film at different ester concentration.

Concentration, %	0.5	1.0	1.5	2.0
∆L*	0.09	1.06	1.39	1.73
∆a*	0.06	0.03	−0.15	−0.35
∆b*	0.86	−0.65	−1.69	−2.22
∆E*_ab_	0.86	1.24	2.19	2.83

**Table 5:** Rheological equations for all bioesters.

Temperature, °C	*η* *_a_* $=k\;{\dot{\gamma }}^{n-1}$	R^2^	n	*η* *_a_* $=k\;{\dot{\gamma }}^{n-1}$	R^2^	n	*η* *_a_* $=k\;{\dot{\gamma }}^{n-1}$	R^2^	n
	B1			M1			C1		
25	*η* *_a_* $=0.1898\;{\dot{\gamma }}^{-0.234}$	0.9191	0.766	*η* *_a_* $=0.1624\;\dot{\gamma }$ ^−0.214^	0.8437	0.786	*η* *_a_* $=0.1670\;\dot{\gamma }$ ^−0.216^	0.8283	0.784
35	*η* *_a_* $=0.1502\;\dot{\gamma }$ ^−0.218^	0.9343	0.782	*η* *_a_* $=0.1594\;\dot{\gamma }$ ^−0.221^	0.9298	0.779	*η* *_a_* $=0.1614\;\dot{\gamma }$ ^−0.221^	0.9269	0.779
50	*η* *_a_* $=0.1644\;\dot{\gamma }$ ^−0.240^	0.9836	0.760	*η* *_a_* $=0.1452\;\dot{\gamma }$ ^−0.220^	0.9511	0.780	*η* *_a_* $=0.1390\;\dot{\gamma }$ ^−0.214^	0.9587	0.786
60	*η* *_a_* $=0.1030\;\dot{\gamma }$ ^−0.198^	0.9740	0.802	*η* *_a_* $=0.0990\;\dot{\gamma }$ ^−0.188^	0.9477	0.812	*η* *_a_* $=0.0936\;\dot{\gamma }$ ^−0.179^	0.9758	0.821
70	*η* *_a_* $=0.0580\;\dot{\gamma }$ ^−0.147^	0.9658	0.853	*η* *_a_* $=0.0550\;\dot{\gamma }$ ^−0.133^	0.9107	0.867	*η* *_a_* $=0.0559\;\dot{\gamma }$ ^−0.133^	0.9542	0.867
	B2			M2			C2		
25	*η* *_a_* $=0.1944\;\dot{\gamma }$ ^−0.229^	0.8779	0.771	*η* *_a_* $=0.1932\;\dot{\gamma }$ ^−0.238^	0.8952	0.762	*η* *_a_* $=0.2029\;\dot{\gamma }$ ^−0.239^	0.8879	0.761
35	*η* *_a_* $=0.1515\;\dot{\gamma }$ ^−0.211^	0.9299	0.789	*η* *_a_* $=0.1520\;\dot{\gamma }$ ^−0.220^	0.9025	0.780	*η* *_a_* $=0.1700\;\dot{\gamma }$ ^−0.228^	0.9348	0.772
50	*η* *_a_* $=0.1400\;\dot{\gamma }$ ^−0.211^	0.9546	0.789	*η* *_a_* $=0.1490\;\dot{\gamma }$ ^−0.227^	0.9594	0.773	*η* *_a_* $=0.1484\;\dot{\gamma }$ ^−0.222^	0.9486	0.778
60	*η* *_a_* $=0.0957\;\dot{\gamma }$ ^−0.179^	0.9668	0.821	*η* *_a_* $=0.0951\;\dot{\gamma }$ ^−0.189^	0.9627	0.811	*η* *_a_* $=0.0968\;\dot{\gamma }$ ^−0.185^	0.9504	0.815
70	*η* *_a_* $=0.0565\;\dot{\gamma }$ ^−0.130^	0.9227	0.870	*η* *_a_* $=0.0608\;\dot{\gamma }$ ^−0.152^	0.9326	0.848	*η* *_a_* $=0.0613\;\dot{\gamma }$ ^−0.147^	0.9351	0.853
	B3			M3			C3		
25	*η* *_a_* $=0.1995\;\dot{\gamma }$ ^−0.223^	0.8926	0.777	*η* *_a_* $=0.2680\;\dot{\gamma }$ ^−0.275^	0.938	0.725	*η* *_a_* $=0.2686\;\dot{\gamma }$ ^−0.268^	0.9327	0.732
35	*η* *_a_* $=0.1482\;\dot{\gamma }$ ^−0.198^	0.9258	0.802	*η* *_a_* $=0.1560\;\dot{\gamma }$ ^−0.225^	0.9111	0.775	*η* *_a_* $=0.1653\;\dot{\gamma }$ ^−0.223^	0.9338	0.777
50	*η* *_a_* $=0.1394\;\dot{\gamma }$ ^−0.200^	0.9502	0.800	*η* *_a_* $=0.1493\;\dot{\gamma }$ ^−0.230^	0.9578	0.770	*η* *_a_* $=0.1375\;\dot{\gamma }$ ^−0.211^	0.9633	0.789
60	*η* *_a_* $=0.0950\;\dot{\gamma }$ ^−0.166^	0.9520	0.834	*η* *_a_* $=0.0956\;\dot{\gamma }$ ^−0.193^	0.9557	0.807	*η* *_a_* $=0.0981\;\dot{\gamma }$ ^−0.184^	0.9595	0.816
70	*η* *_a_* $=0.0575\;\dot{\gamma }$ ^−0.120^	0.9321	0.880	*η* *_a_* $=0.0646\;\dot{\gamma }$ ^−0.161^	0.9281	0.839	*η* *_a_* $=0.0584\;\dot{\gamma }$ ^−0.138^	0.9284	0.862

**Table 6:** Particular types of the Arrhenius equation for all esters.

$\dot{\gamma }$	*η_a_ = Aʹ* × 10^3^ *∙* exp*^Ea/RT^*	E_a_ (kJ/mol)	*η_a_ = Aʹ* × 10^3^ *∙* exp*^Ea/RT^*	E_a_ (kJ/mol)	*η_a_ = Aʹ ×* 10^3^ *∙* exp*^Ea/RT^*	E_a_ (kJ/mol)
	B1		M1		C1	
4000	*η_a_* = 0.66 ∙ exp^1130.7/T^	9.4	*η_a_* = 0.90 ∙ exp^1045.1/T^	8.7	*η_a_* = 0.83 ∙ exp^1074.8/T^	8.9
9333	*η_a_* = 1.19 ∙ exp^886.2/T^	7.4	*η_a_* = 1.92 ∙ exp^754.8/T^	6.3	*η_a_* = 1.62 ∙ exp^809.2/T^	6.7
13,333	*η_a_* = 1.84 ∙ exp^696.6/T^	5.8	*η_a_* = 2.50 ∙ exp^610.8/T^	5.1	*η_a_* = 3.22 ∙ exp^535.6/T^	4.4
	B2		M2		C2	
4000	*η_a_* = 0.97 ∙ exp^1030.8/T^	8.6	*η_a_* = 0.82 ∙ exp^1057.4/T^	8.8	η_a_ = 0.77 ∙ exp^1093.4/T^	9.1
9333	*η_a_* = 1.98 ∙ exp^757.8/T^	6.3	*η_a_* = 1.19 ∙ exp^891.9/T^	7.4	*η_a_* = 1.52 ∙ exp^813.0/T^	6.9
13,333	*η_a_* = 3.18 ∙ exp^550.5/T^	4.6	*η_a_* = 2.04 ∙ exp^659.4/T^	5.5	*η_a_* = 2.35 ∙ exp^629.0/T^	5.2
	B3		M3		C3	
4000	*η_a_* = 1.38 ∙ exp^947.4/T^	7.9	*η_a_* = 0.66 ∙ exp^1127.9/T^	9.4	*η_a_* = 0.87 ∙ exp^1059.9/T^	8.8
9333	*η_a_* = 2.52 ∙ exp^710.8/T^	5.9	*η_a_* = 1.24 ∙ exp^872.6/T^	7.2	*η_a_* = 1.91 ∙ exp^759.2/T^	6.3
13,333	*η_a_* = 3.88 ∙ exp^519.8/T^	4.3	*η_a_* = 1.68 ∙ exp^711.9/T^	5.9	*η_a_* = 3.00 ∙ exp^559.1/T^	4.6

## Data Availability

All the experimental data obtained are presented, in the form of table and/or figure, in the article.

## References

[1] Mosoarca G., Negrea A. (2012). Studies regarding the effects of settling tanks sludge recycling on organic matter concentration from treated water. J. Environ. Prot. Ecol..

[2] Syamsuddin Y., Murat M.N., Hameed B.H. (2016). Synthesis of fatty acid methyl ester from the transesterification of high- and low-acid-content crude palm oil (*Elaeis guineensis*) and karanj oil (*Pongamia pinnata*) over a calcium-lanthanum-aluminum mixed-oxides catalyst. Bioresour. Technol..

[3] Toscano G., Duca D. (2009). Renewable energy content of fatty acid methyl esters (fame) and glycerol. J. Agric. Eng..

[4] Amani H., Asif M., Hameed B.H. (2016). Transesterification of waste cooking palm oil and palm oil to fatty acid methyl ester using cesium-modified silica catalyst. J. Taiwan Inst. Chem..

[5] Stacy C.J., Melick C.A., Cairncross R.A. (2014). Esterification of free fatty acids to fatty acid alkyl esters in a bubble column reactor for use as biodiesel. Fuel Process. Technol..

[6] Ye W., Gao Y., Ding H., Liu M., Liu S., Han X., Qi J. (2016). Kinetics of transesterification of palm oil under conventional heating and microwave irradiation, using CaO as heterogeneous catalyst. Fuel.

[7] Oh J., Yang S., Kim C., Choi I., Kim J.H. (2013). Synthesis of biolubricants using sulfated zirconia catalysts. Appl. Catal. A-Gen..

[8] Hamadou B., Djomdi, Falama R.Z., Delattre C., Pierre G., Dubessay P., Michaud P. (2020). Influence of Physicochemical Characteristics of Neem Seeds (Azadirachta indica A. Juss) on Biodiesel Production. Biomolecules.

[9] Sanchez N., Encinar J.M., Nogales S., González J.F. (2019). Biodiesel Production from Castor oil by Two-Step Catalytic Transesterification: Optimization of the Process and Economic Assessment. Catalysts.

[10] Ramkumar S., Kirubakaran V. (2016). Biodiesel from vegetable oil as alternate fuel for C.I engine and feasibility study of thermal cracking: A critical review. Energy Convers. Manag..

[11] Cursaru D., Neagu M., Bogatu L. (2013). Investigations on the oxidation stability of biodiesel synthesized from different vegetable oils. Rev. Chim..

[12] Dos Santos P.R.S., Voll F.A.P., Ramos L.P., Corazzaa M.L. (2017). Esterification of fatty acids with supercritical ethanol in a continuous tubular reactor. J. Supercrit. Fluids.

[13] Gude V.G., Patil P., Martinez-Guerra E., Deng S., Nirmalakhandan N. (2013). Microwave energy potential for biodiesel production. Sustain. Chem. Process..

[14] Ilia G., Simulescu V., Mak C.A., Crasmareanu E. (2014). The use of transesterification method for obtaining phosphorus-containing polymers. Adv. Polym. Technol..

[15] Monroe E., Somnath S., Carlson J.S., Eckles T.P., Liu F., Varman A.M., George A., Davis R.W. (2020). Superior performance biodiesel from biomass-derived fusel alcohol and low grade oils: Fatty acid fusel esters (FAFE). Fuel.

[16] Popa S., Milea M.S., Boran S., Nitu S.V., Mosoarca G., Vancea C., Lazau R.I. (2020). Rapid adulteration detection of cold pressed oils with their refined versions by UV-VIS spectroscopy. Sci. Rep..

[17] Radulescu M.E., Visa A., Milea M.S., Lazau R.I., Popa S., Funar-Timofei S. (2020). Synthesis, spectral characterization, and theoretical investigations of a new azo-stilbene dye for acrylic resins. J. Mol. Struct..

[18] Wozniak M., Kowalska M., Tavernier S., Zbikowska A. (2021). Enzymatically Modified Fats Applied in Emulsions Stabilized by Polysaccharides. Biomolecules.

[19] Umerov S.O., Dulina I.O., Ragulya A.V. (2015). Rheology of plasticized polymer solutions. J. Silicate Based Compos. Mater..

[20] Ahmed W.A., Yarmo A., Salih N., Derawi M.D., Yusop M.R., Salimon J. (2015). Synthesis and lubricity properties analysis of branched dicarboxylate esters based lubricant. Malaysian J. Anal. Sci..

[21] Attia N.K., El-Mekkawi S.A., Elardy O.A., Abdelkader E.A. (2020). Chemical and rheological assessment of produced biolubricants from different vegetable oils. Fuel.

[22] Dardan E., Afrand M., Isfahani A.H.M. (2016). Effect of suspending hybrid nano-additives on rheological behavior of engine oil and pumping power. Appl. Therm. Eng..

[23] Nogales-Delgado S., Encinar J.M., Cortes A.G. (2021). High oleic safflower oil as a feedstock for stable biodiesel and biolubricant production. Ind. Crops Prod..

[24] Owuna F.J., Dabai M.U., Sokoto M.A., Dangoggo S.M., Bagudo B.U., Birnin-Yauri U.A., Hassan L.G., Sada I., Abubakar A.L., Jibrin M.S. (2020). Chemical modification of vegetable oils for the production of biolubricants using trimethylolpropane: A review. Egyp. J. Pet..

[25] Petrova V.A., Elokhovskiy V.Y., Raik S.V., Poshina D.N., Romanov D.P., Skorik Y.A. (2019). Alginate Gel Reinforcement with Chitin Nanowhiskers Modulates Rheological Properties and Drug Release Profile. Biomolecules.

[26] Vulpe R., Le Cerf D., Dulong V., Popa M., Peptu C., Verestiuc L., Picton L. (2016). Rheological study of in-situ crosslinkable hydrogels based on hyaluronanic acid, collagen and sericin. Mat. Sci. Eng. C-Mater..

[27] Helminen A., Kylma J., Tuominen J., Seppala J.V. (2000). Effect of structure modification on rheological properties of biodegradable poly(ester-urethane). Polym. Eng. Sci..

[28] Staroszczyk H., Fiedorowicz M., Opalinska-Piskorz J., Tylingo R. (2013). Rheology of potato starch chemically modified with microwave-assisted reactions. LWT-Food Sci. Technol..

[29] Simulescu V., Crasmareanu E., Ilia G. (2011). Synthesis, properties and structures of phosphorus-nitrogen heterocycles. Heterocycles.

[30] Wong C.P., Berry G.C. (1979). Rheological studies on concentrated solutions of heterocyclic polymers. Polymer.

[31] Yin C.Q., Dong J., Zhang Z.X., Zhang Q.H. (2014). Rheological behaviour of polyamic acid spinning solutions containing heterocycle. Mater. Res. Innov..

[32] Sharma A.K., Tiwari A.K., Dixit A.R. (2016). Rheological behaviour of nanofluids: A review. Renew. Sust. Energ. Rev..

[33] Shu R., Gan Y., Lv H., Tan D. (2016). Preparation and rheological behavior of ethylene glycol-based TiO_2_ nanofluids. Colloid Surf. A Physicochem. Eng. Asp..

[34] Popa S., Boran S., Simulescu V. (2017). Collagen films obtained from collagen solutions characterized by rheology. Mater. Plast..

[35] Hoti G., Caldera F., Cecone C., Rubin Pedrazzo A., Anceschi A., Appleton S.L., Khazaei Monfared Y., Trotta F. (2021). Effect of the Cross-Linking Density on the Swelling and Rheological Behavior of Ester-Bridged β-Cyclodextrin Nanosponges. Materials.

[36] Bokhari A., Yusup S., Chuah L.F., Kamil R.N.M. (2016). Relative efficiency of esterified rubber seed oil in a hydrodynamic cavitation reactor and purification via distillation column. Chem. Eng. Trans..

[37] Kuzmina J.S., Director L.B., Shevchenko A.L., Zaichenko V.M. (2016). Energy efficiency analysis of reactor for torrefaction of biomass with direct heating. J. Phys. Conf. Ser..

[38] Encinar J.M., Gonzalez J.F., Martinez G., Sanchez N., Pardal A. (2012). Soybean oil transesterification by the use of a microwave flow system. Fuel.

[39] Kazimierowicz J., Zieliński M., Dębowski M. (2021). Influence of the Heating Method on the Efficiency of Biomethane Production from Expired Food Products. Fermentation.

[40] Yu B.-S., Hung W.-H., Fang J.-N., Yu Y.-T. (2020). Synthesis of Zn-Saponite Using a Microwave Circulating Reflux Method under Atmospheric Pressure. Minerals.

[41] Wardhono E.Y., Wahyudi H., Agustina S., Oudet F., Pinem M.P., Clausse D., Saleh K., Guénin E. (2018). Ultrasonic Irradiation Coupled with Microwave Treatment for Eco-friendly Process of Isolating Bacterial Cellulose Nanocrystals. Nanomaterials.

[42] Zieliński M., Dębowski M., Kazimierowicz J. (2021). Microwave Radiation Influence on Dairy Waste Anaerobic Digestion in a Multi-Section Hybrid Anaerobic Reactor (M-SHAR). Processes.

[43] Zimmermann K. (2018). Microwave Technologies: An Emerging Tool for Inactivation of Biohazardous Material in Developing Countries. Recycling.

[44] Zulqarnain, Ayoub M., Yusoff M.H.M., Nazir M.H., Zahid I., Ameen M., Sher F., Floresyona D., Budi Nursanto E. (2021). A Comprehensive Review on Oil Extraction and Biodiesel Production Technologies. Sustainability.

[45] Popa S., Mosoarca G., Macarie L., Plesu N., Ilia G., Tara-Lunga-Mihali M. (2021). Copolymerization of butyl acrylate with methyl methacrylate in a bubble column reactor and the use of copolymer in corrosion protection. Polym. Bull..

[46] Dange P.N., Rathod V.K. (2017). Equilibrium and thermodynamic parameters for heterogeneous esterification of butyric acid with methanol under microwave irradiation. Resour. Efficient. Technol..

[47] Popa S., Boran S. (2016). Energetic efficiency calculation for a new experimental reactor. Mater. Plast..

[48] Winkler H., Vorwerg W., Rihm R. (2014). Thermal and mechanical properties of fatty acid starch esters. Carbohydr. Polym..

[49] Paul A.K., Achar S.K., Dasari S.R., Borugadda V.B., Goud V.V. (2017). Analysis of thermal, oxidative and cold flow properties of methyl and ethyl esters prepared from soybean and mustard oils. J. Therm. Anal. Calorim..

[50] Sari A., Karaipekli A., Eroglu R., Biçer A. (2013). Erythritol Tetra Myristate and Erythritol Tetra Laurate as Novel Phase Change Materials for Low Temperature Thermal Energy Storage. Energ. Source Part A.

[51] Hu C., Ai J., Ma L., Wen P., Fan M., Zhou F., Liu W. (2021). Ester Oils Prepared from Fully Renewable Resources and Their Lubricant Base Oil Properties. ACS Omega.

[52] Nakayama Y., Watanabe K., Tanaka R., Shiono T., Kawasaki N., Yamano N., Nakayama A. (2020). Synthesis, Properties, and Biodegradation of Sequential Poly(Ester Amide)s Containing γ-Aminobutyric Acid. Int. J. Mol. Sci..

[53] Aziz N.A.M., Yunus R., Hamid H.A., Ghassan A.A.K., Omar R., Rashid U., Abbas Z. (2020). An acceleration of microwave-assisted trans-esterification of palm oil-based methyl ester into trimethylolpropane ester. Sci. Rep..

[54] Veronesi F., Guarini G., Corozzi A., Raimondo M. (2021). Evaluation of the Durability of Slippery, Liquid-Infused Porous Surfaces in Different Aggressive Environments: Influence of the Chemical-Physical Properties of Lubricants. Coatings.

[55] Zhang X., Li G., Chen Y., Wang K., Yang E. (2021). The Synthesis of Associative Copolymers with Both Amphoteric and Hydrophobic Groups and the Effect of the Degree of Association on the Instability of Emulsions. Polymers.

[56] Soares I.P., Rezende T.F., Silva R.C., Castro E.V.R., Fortes I.C.P. (2008). Multivariate calibration by variable selection for blends of raw soybean oil/biodiesel from different sources using Fourier Transform Infrared spectroscopy (FTIR) spectra data. Energy Fuels.

[57] Duan Y., Huo Y., Duan L. (2017). Preparation of acrylic resins modified with epoxy resins and their behaviors as binders of waterborne printing ink on plastic film. Colloids Surf. A Physicochem. Eng. Asp..

[58] Jiao C., Sun L., Shao Q., Song J., Hu Q., Naik N., Guo Z. (2021). Advances in Waterborne Acrylic Resins: Synthesis Principle, Modification Strategies, and Their Applications. ACS Omega.

[59] Hajipour A., Shams-Nateri A. (2021). Expanding the color gamut of inkjet textile printing during color matching. Color Res. Appl..

[60] Popa S., Radulescu-Grad M.E., Perdivara A., Mosoarca G. (2021). Aspects regarding colour fastness and adsorption studies of a new azo-stilbene dye for acrylic resins. Sci. Rep..

[61] Golshan M., Amani F., Salami-Kalajahi M. (2021). Photophysical and reflectance properties of perylene-3,4,9,10-tetracarboxylic diimide (PTCDI)/rhodamine 6 G hybrid for application in cold paints. Prog. Org. Coat..

[62] Che J. (2018). Relative analysis of the dependency of spectrophotometric data on temperature using textiles, ceramics, plastics, paints, and printed materials. Color Res. Appl..

[63] Kowalska M., Turek P., Zbikowska A., Babut M., Szakiel J. (2021). The Quality of Emulsions with New Synthetized Lipids Stabilized by Xanthan Gum. Biomolecules.

[64] Boran S., Tamas A., Mosoarca G. (2019). Soybean bioester obtained in a bubble column esterification reactor—A rheological study. Studia UBB Chemia.

[65] Ladie R., Cosentino C., Tagliaro I., Antonini C., Bianchini G., Bertini S. (2021). Supramolecular Structuring of Hyaluronan-Lactose-Modified Chitosan Matrix: Towards High-Performance Biopolymers with Excellent Biodegradation. Biomolecules.

[66] Sienkiewicz A., Czub P. (2021). Rheological Analysis of the Synthesis of High-Molecular-Weight Epoxy Resins from Modified Soybean Oil and Bisphenol A or BPA-Based Epoxy Resins. Materials.

[67] Ahmad M.N., Ishak M.R., Taha M.M., Mustapha F., Leman Z. (2021). Rheological and Morphological Properties of Oil Palm Fiber-Reinforced Thermoplastic Composites for Fused Deposition Modeling (FDM). Polymers.

